# Central Circadian Control of Female Reproductive Function

**DOI:** 10.3389/fendo.2013.00195

**Published:** 2014-01-22

**Authors:** Brooke H. Miller, Joseph S. Takahashi

**Affiliations:** ^1^Departments of Psychiatry and Medicine, University of Florida College of Medicine, Gainesville, FL, USA; ^2^Department of Neuroscience, Howard Hughes Medical Institute, University of Texas Southwestern Medical Center, Dallas, TX, USA

**Keywords:** circadian rhythms, estrous cycle, clock gene, proestrus, parturition

## Abstract

Over the past two decades, it has become clear just how much of our physiology is under the control of the suprachiasmatic nucleus (SCN) and the cell-intrinsic molecular clock that ticks with a periodicity of approximately 24 h. The SCN prepares our digestive system for meals, our adrenal axis for the stress of waking up in the morning, and the genes expressed in our muscles when we prepare to exercise. Long before molecular studies of genes such as *Clock, Bmal1*, and the *Per* homologs were possible, it was obvious that female reproductive function was under strict circadian control at every level of the hypothalamic-pituitary-gonadal axis, and in the establishment and successful maintenance of pregnancy. This review highlights our current understanding of the role that the SCN plays in regulating female reproductive physiology, with a special emphasis on the advances made possible through the use of circadian mutant mice.

## Introduction to the Regulation of Female Reproduction by the Central Circadian System

The light:dark cycle is the most predictable environmental cue available to animals, and determines basic environmental factors such as food and mate availability and predator/prey dynamics. Organisms have therefore developed a reliable biological mechanism to anticipate the changes in their environment, and to adjust their behavior and physiology appropriately. In mammals, the biological basis of this predictor is a cell-intrinsic molecular circadian pacemaker within the suprachiasmatic nuclei (SCN) of the hypothalamus. At its most basic level, the molecular circadian clock is composed of a transcription-translation loop: two proteins, CLOCK and BMAL1 (also known as MOP3), induce the transcription of four other genes, *Per1, Per2, Cry1*, and *Cry2*, which, after translation, dimerize, re-enter the nucleus, and inhibit their own transcription (1, 2). Following turnover of the inhibitory proteins, the transcription-translation cycle resumes. This molecular cycle occurs over a period of approximately 24 h and, in the absence of any external input, the neuronal network within the SCN maintains a stable period almost indefinitely. All tissues contain this basic molecular clock, but, in general the SCN act as the master pacemaker in a hierarchical system of multiple oscillators, receiving time-of-day input from photosensitive melanopsin-containing retinal ganglion cells, and phase-coordinating the activity of peripheral tissue-specific oscillators via neuronal and humoral output (3, 4).

This review will primarily focus on the female rodent, the most well-studied model of circadian reproductive neuroendocrinology. In the female, reproductive function is dependent on a tightly orchestrated cascade of events that originate from gonadotropin-releasing hormone (GnRH) neurons distributed throughout the preoptic area (POA) and septal areas of the basal forebrain. On the afternoon of proestrus, the stage prior to ovulation, a surge of GnRH released from neuronal terminals in the mediobasal hypothalamus induces the pituitary to release a timed bolus of luteinizing hormone (LH) and follicle stimulating hormone (FSH), which act on the ovary to induce ovulation and follicular recruitment. Regulation of the preovulatory GnRH surge itself is primarily controlled by two types of input to GnRH neurons and their surrounding afferent neurons: estradiol feedback from maturing ovarian follicles, and time-of-day output from the suprachiasmatic nucleus (SCN). The absence or dysregulation of either type of input disrupts GnRH surge release and results in an anovulatory cycle (5–8).

Although proper levels of estrogen and progesterone create a permissive state for GnRH release, a timing signal from the SCN is required to induce the level of GnRH release associated with the LH surge. The signal from the SCN to GnRH neurons occurs once daily: if rats are treated chronically with high levels of estradiol, an LH surge can be observed on multiple successive days, always restricted to the late afternoon (9). Even in the presence of high estradiol levels, ablation of the SCN or disruption of the neuronal projections from the SCN to the POA, including kisspeptin-secreting neurons, results in estrous acyclicity, indicating the crucial role the SCN play in reproductive function (10–13).

In addition to the estrous cycle, certain aspects of pregnancy are also under circadian control. The mating stimulus triggers a series of daily, biphasic surges in prolactin (PRL) occurring during the early morning and early evening hours (14). Knife cuts to the retrochiasmatic region prevent circadian prolactin release during pseudopregnancy (14) and prolactin surges during the estrous cycle, indicating that PRL release is SCN-dependent (15). Parturition is also under circadian regulation; although the major contributory factors to the onset of parturition vary among species, the timing of the onset of parturition generally occurring during the species-dependent inactive phase (e.g., day-time for nocturnal rodents and night-time for diurnal humans) (16).

Reproductive function can be disrupted by interfering with many of the core genetic components of the molecular pacemaker, including *Clock* (17), *Bmal1* (18), and *Per1* and *Per2* (19). Recent studies of reproduction in circadian mutant mice have brought a new dimension of molecular specificity to our understanding of central circadian control over reproductive endocrinology. Here, we review the current literature regarding the role of the SCN in regulating female estrous cycles, the establishment and maintenance of pregnancy, and parturition. The importance of circadian rhythms in peripheral reproductive tissue is reviewed elsewhere in this issue.

## The Role of the SCN in the Regulation of the Estrous Cycle

**Figure 1:**
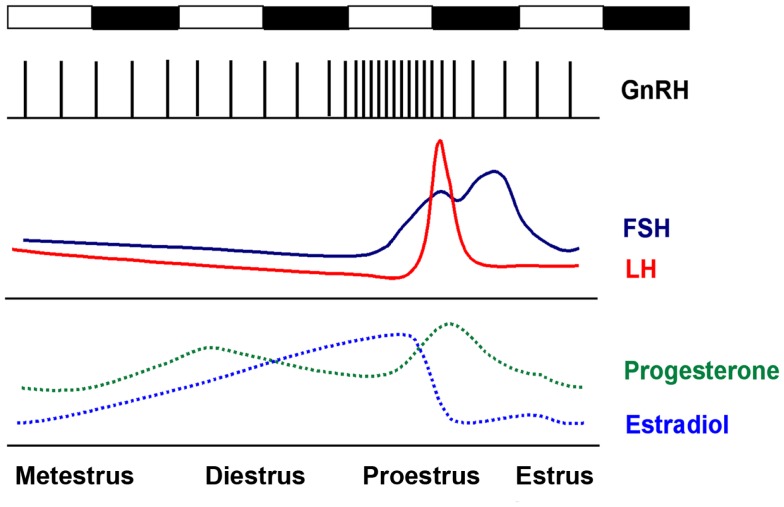
**The rodent estrous cycle**. In the mouse, ovulation occurs every 4–5 days. Metestrus and diestrus are characterized by low but slowly increasing levels of estradiol. On the late afternoon of proestrus, elevated estradiol levels induce a bolus of GnRH release from the hypothalamus, which induces the proestrus LH and FSH surge at approximately the start of the active (dark) period. Ovulation occurs 12–14 h later.

Ovulation in the female mammal is a complex and carefully timed process that is governed both centrally, by GnRH neurons in the hypothalamus, and locally, by hormones released by cells proximate to the maturing follicle; these same hormones modulate GnRH neuron activity (Figure 1). The first two stages of the cycle, metestrus and diestrus, are characterized by low levels of estradiol, as follicles recruited during the previous estrous cycle progress from the early antral to preovulatory stage. On late diestrus and early proestrus, serum levels of estradiol, produced primarily by the granulosa cells in the developing preovulatory (Graafian) follicles, peak. Elevated estradiol induces a number of molecular and morphological changes in GnRH neurons and the interneurons that surround and modulate GnRH neurons, including the upregulation of progesterone receptors and an increase in the amplitude and frequency of GnRH pulsatility (20–22). The end result of estradiol priming is a surge in GnRH release on late proestrus, the precise timing of which is gated by a neurally derived timing signal. The proestrus GnRH surge induces release of LH and FSH from gonadotropes in the pituitary; in the ovary, LH induces ovulation of Graafian follicles, while FSH stimulates the recruitment of a new cohort of antral follicles. In the mouse and rat, estrous cycles normally last 4–5 days.

Elevated estradiol is mandatory for a GnRH surge to occur. It is not, however, sufficient. More than 50 years ago, it was shown that the preovulatory GnRH surge is controlled by two types of input to GnRH neurons and their surrounding interneurons: hormonal feedback from maturing ovarian follicles and a time-restricted neuronal signal (23). Everett and Sawyer demonstrated that female rats treated with pentobarbital at 1400 h, but not 1600 h, exhibit a 24-h delay in both the LH surge and ovulation. Bingel and Schwartz later demonstrated a similar effect of proestrus pentobarbital treatment on ovulation in the mouse (24). Both groups determined that the effect of the pentobarbital was to inhibit the then-unknown signal driving the LH surge and subsequent ovulation. It was later shown that the neural timing signal occurs once daily: if rats are treated chronically with high levels of estradiol, an LH surge can be observed on multiple successive days, always restricted to the late afternoon (9, 25).

The SCN was identified as the locus of the neural timing signal when it was shown that ablating the SCN, or severing neuronal connections between the SCN and the POA, resulted in estrous acyclicity (10–12). Tract-tracing and immunohistochemical studies have identified direct SCN-GnRH neuron connections, and indirect connections in which SCN neurons synapse on estradiol-concentrating interneurons adjacent to GnRH neurons in the anterior POA (26, 27). The SCN-derived signal is believed to be neural, rather than humoral, in nature: when a SCN-lesioned females hamster receives a transplanted SCN that is capable of sustaining molecular rhythms but does not form normal neural connections, certain rhythms, such as locomotor activity, are restored, but estrous cyclicity is not (28–30).

Gonadotropin-releasing hormone neurons represent the final point of convergence between the hormonal and timing signals, but also possess an intrinsic circadian clock that regulates GnRH transcription, translation, and release. Several groups have shown that core clock genes, including *Bmal1* and the *Period* genes, are expressed and cycle rhythmically in both GnRH neurons and the immortalized GT1-7 GnRH neuronal cell line (31, 32). Dysregulation of certain circadian genes, such as *Clock* and *Cry1*, disrupts ultradian GnRH pulse amplitude and frequency (33), and the effect of the potent GnRH secretagogue kisspeptin is significantly reduced in preoptic explants from *Bmal1* knockout mice (34).

Although the precise nature of the timing signal is still under debate, both vasopressin (AVP) and vasoactive intestinal polypeptide (VIP) appear to regulate the timing of GnRH release. Both polypeptides are rhythmically expressed and present in SCN neurons efferent to the mPOA, and, in rats, inhibition of either AVP or VIP signaling results in a reduction in the amplitude of an estradiol-induced LH surge (35–37), and VIP can regulate the timing of the LH surge within a specific timeframe (38). However, AVP alone is capable of inducing an LH surge in SCN-lesioned rats, and if the rhythms of AVP and VIP are phase-dissociated in SCN-POA co-cultures, AVP secretion occurs in phase with GnRH release, whereas VIP secretion does not (39, 40).

**Figure 2:**
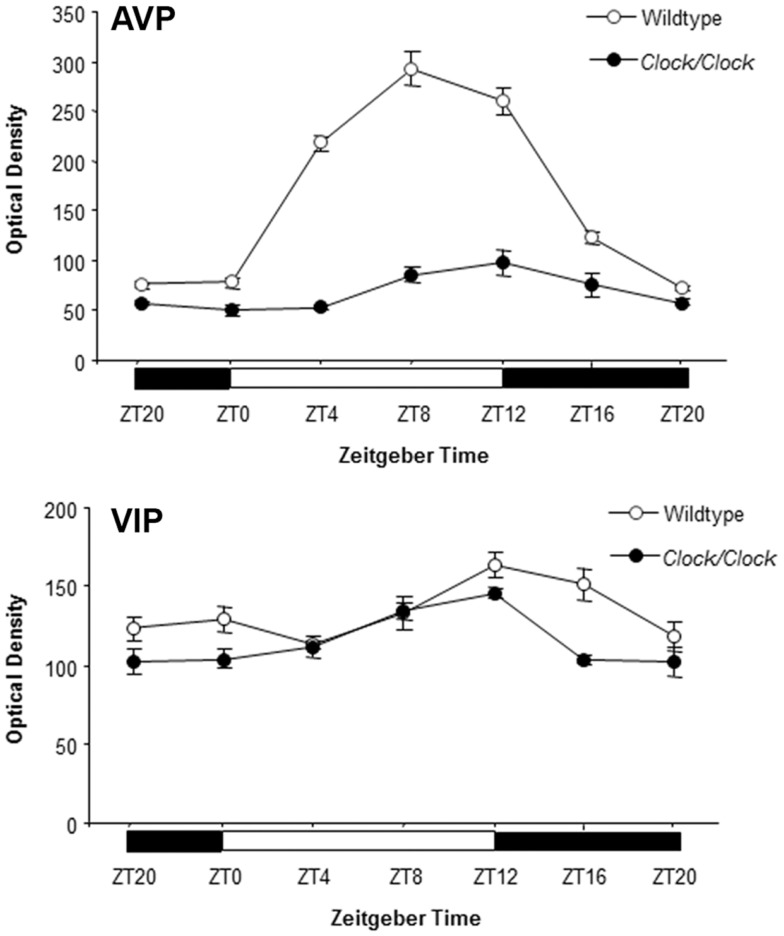
**AVP and VIP expression in the SCN**. AVP and VIP mRNA levels were measured by autoradiography in the SCN from wildtype and *Clock/Clock* mice. Both AVP and VIP showed circadian expression in wildtype SCN. In *Clock* mutants, VIP expression was similar to that observed in wildtype mice, but AVP expression and rhythmicity was significantly damped. Modified from data presented in Ref. (43).

Genetic mouse models have allowed for a more precise understanding of the timing signal. Expression of both AVP and its receptor, *V1aR*, are reduced in the SCN of both male and female mice that are homozygous for the CLOCK-Δ19 dominant negative mutation, whereas VIP expression is not affected by the *Clock* mutation (Figure 2). Moreover, AVP injection into the region of the medial POA on the afternoon of proestrus is sufficient to rescue the LH surge in *Clock/Clock* mutant female mice (41–43). The effect of AVP is time dependent: administration of AVP in the afternoon, but not morning, of proestrus induces LH release (44).

**Figure 3:**
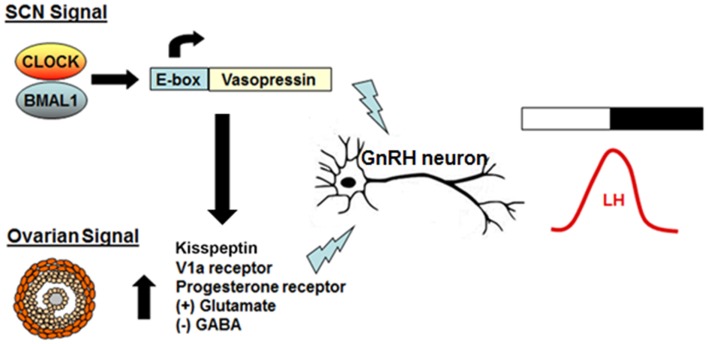
**Model of the interaction between the daily timing signal and the ovarian signal on GnRH release**. AVP is rhythmically transcribed in the SCN and forms the basis for the strong neural daily timing signal from the SCN. On the day of proestrus, estradiol produced by the developing follicles in the ovary upregulates expression of hypothalamic AVP and VIP receptors and primes kisspeptin neurons to become more sensitive to the SCN-derived signal. The end result is an increase in excitatory input to GnRH neurons, elevated GnRH release, and a subsequent surge in LH.

Neurons expressing the GnRH secretagogue kisspeptin, which has been shown to play a critical role in the onset of puberty, also may control the timing of the proestrus GnRH surge. Mice lacking either *Kiss1* or its receptor, *GPR54*, generally do not have an LH surge, and the circadian expression of *Kiss1* and *c-fos* in KISS1-secreting neurons is dependent on the presence of estrogen (45–47). *In vitro* and *in vivo* experiments have shown that elevated estradiol levels produce circadian expression of the kisspeptin peptide receptor GPR54 (48). Additionally, Williams and colleagues showed that *Kiss1R* neurons are targeted by AVP neurons from the SCN, and that there is a strong circadian rhythm in the response of GnRH neurons to kisspeptin, suggesting that Kisspeptin neurons may represent the locus of integration of the estradiol and circadian signals (49). In this model, rising estradiol levels on proestrus upregulate factors that promote GnRH release, while daily rhythmic expression of AVP gates the timing of LH release via direct and indirect regulation of GnRH neurons (50, 51) (Figure 3).

**Figure 4:**
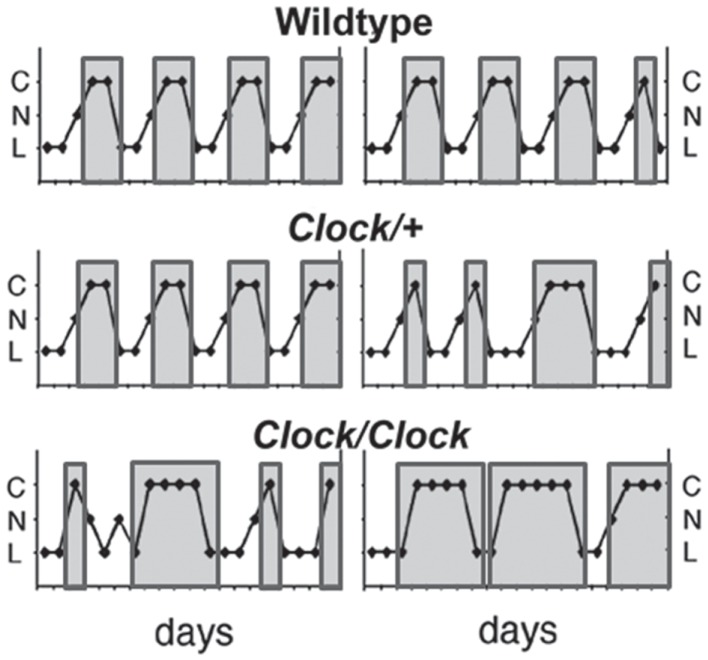
***Clock/Clock* females display lengthened and irregular estrous cycles**. Representative estrous cycles as measured by vaginal cytology from wildtype (top), *Clock*/+ (middle), and *Clock/Clock* (bottom) females. *Clock/Clock* females have significantly fewer days of nucleated (proestrus) smears and significantly more days of cornified (estrus) smears compared to wildtype females. C, cornified; N, nucleated; L, leukocytic. Modified from a figure in Ref. (17).

In addition to disruption or ablation of the LH surge in circadian mutant mice, the estrous cycle itself is often affected. Multiple groups have shown that *Clock/Clock* mutant mice have an extended and disrupted estrous cycle under both light:dark and continuous darkness, although this effect is somewhat dependent on strain background due to genetic variations in the recently identified CLOCK-Δ19 phenotype suppressor gene *Usf1* (17, 52, 53) (Figure 4). Deletion of the CLOCK binding partner *Bmal1* similarly results in prolonged estrous cycles (18), and although deletion of either *Per1* or *Per2* does not affect the estrous cycle in young mice, it accelerates age-related changes in the estrous cycle (19). Surprisingly, double knockout of either the *Per* or *Cry* paralogs has not been reported to affect estrous cyclicity, despite causing behavioral arrhythmicity in complete darkness (54, 55). These data suggest that CLOCK and BMAL1 have extra-circadian roles in regulating progression of the estrous cycle.

## The Role of the SCN and Circadian Rhythms in the Establishment and Maintenance of Pregnancy

Ovulation takes place on the early morning of estrus, approximately 12 h after the proestrus LH surge (24). If mating occurs, the copulatory stimulus induces the initiation of a pattern of biphasic circadian prolactin surges: a low-amplitude diurnal surge and a high-amplitude nocturnal surge (56). Regulation of PRL release is under SCN control, likely via inhibitory VIP output from the SCN to the dopaminergic neurons in the periventricular-arcuate nucleus (PVN) of the hypothalamus that generally suppress PRL release from the pituitary (57, 58). In non-pregnant females, VIP and PRL expression in the hypothalamus are phase-linked, dopamine neurons in the PVN express the VIP receptor VPAC2, and disruption of VIP can block circadian PRL release (59).

Pituitary prolactin release occurs for 10 days following a fertile mating, or for 12 days following a sterile mating (pseudopregnancy), the difference dependent on the presence of placental lactogen (PL), a prolactin-like hormone produced by the placenta that assumes control of the corpus luteum (CL) while inhibiting maternal prolactin secretion (14). The primary function of prolactin release appears to be to rescue and support the progesterone-producing CL for the first half of pregnancy (60). Placental production of PL is initiated on day 8–9 of pregnancy and continues through the end of pregnancy (61). Maintenance of the CL is an absolute requirement through late pregnancy: if CL maintenance is disrupted and progesterone levels drop, the developing fetuses are reabsorbed (60).

Placental PL production peaks at approximately day 14 of pregnancy and then declines, a pattern that parallels that of progesterone released from the CL (62). The precipitous decline in circulating progesterone levels relieves the inhibition of myometrial contractility, allowing parturition to progress. The drop in progesterone as fetuses near term is critical to successful labor: although estradiol levels increase in the final 24–48 h of pregnancy and estradiol plays an important role in the initiation of parturition, neither estradiol nor oxytocin treatment can overcome the inhibitory effects of elevated progesterone levels on uterine contractions (63).

Despite extensive observational data indicating a role for the central pacemaker in pregnancy, the molecular effects of circadian rhythms on pregnancy have received less attention than their effects on the estrous cycle and LH surge. However, *Clock* mutant mice and *Bmal1, Per1*, and *Per2* knockout mice have all be shown to have deficiencies in embryonic implantation, maintenance of pregnancy, and/or parturition. In middle-aged female mice, deletion of either *Per1* or *Per2* results in an increase in the rate of fetal reabsorption (19), although this effect is not apparent in younger mice. *Bmal1* knockout mice ovulate, but exhibit poor corpora luteum formation, reduced progesterone synthesis, and a complete lack of embryonic implantation, and it is not known whether this is due to a failure of embryonic development or of implantation (64, 65).

Here, comprehensive evaluation of the progression of pregnancy in *Clock/Clock* mice is instructive of the periods during pregnancy that are most sensitive to circadian disruption. *Clock/Clock* mutant females have been shown to have difficulties at multiple stages of pregnancy. Despite a reduced or absent proestrus LH surge, ovaries from non-pregnant homozygous mutant mice contain similar numbers of corpora lutea as wildtype mice (17), suggesting that ovulation, when it occurs, is generally normal. However, the duration of pseudopregnancy, an indirect measure of prolactin release, is significantly shortened in *Clock/Clock* mutants, as would be expected by recent data showing that inhibition of several core clock genes in the SCN can prevent the onset of cervical stimulation-induced biphasic PRL release (58). Indeed, the rate of homozygous mutants that become pregnant is significantly less than half that of wildtype mice, but the number of implanted fetuses at 11 days post-copulation (dpc) is the same in both genotypes. Thus, in *Clock/Clock* females that do become pregnant, the early processes, including embryonic development and implantation, appear normal.

**Figure 5:**
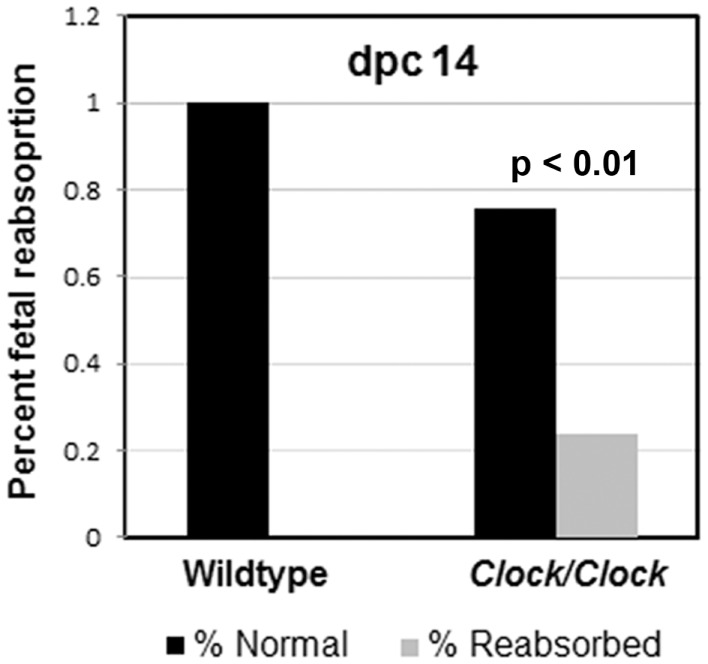
***Clock/Clock* pregnancies exhibit abnormal fetal reabsorption**. In *Clock* mutants that do become pregnant, the early stages of pregnancy, including ovulation, implantation, and embryonic development appear normal. However, by mid-pregnancy, *Clock/Clock* females exhibit a significant increase in fetal reabsorption. Modified from data presented in Ref. (17).

However, *Clock/Clock* mutant serum progesterone levels are substantially lower at dpc 11, and significantly increased fetal reabsorption is observed by dpc 14, and more so at dpc 17 (Figure 5) (17). High midgestational (dpc 11–14) levels of progesterone are particularly important for maintaining blood flow to developing fetuses (66), and previous studies have shown a quantitative relationship between progesterone levels and maintenance of pregnancy (67). The low levels of progesterone in the *Clock* mutants at dpc 11 is the most likely explanation for increased pup reabsorption by mid-pregnancy; although PRL levels were not measured in these animals, the close association between the SCN and PRL release suggest a failure of maternal PRL release at the central level.

**Figure 6:**
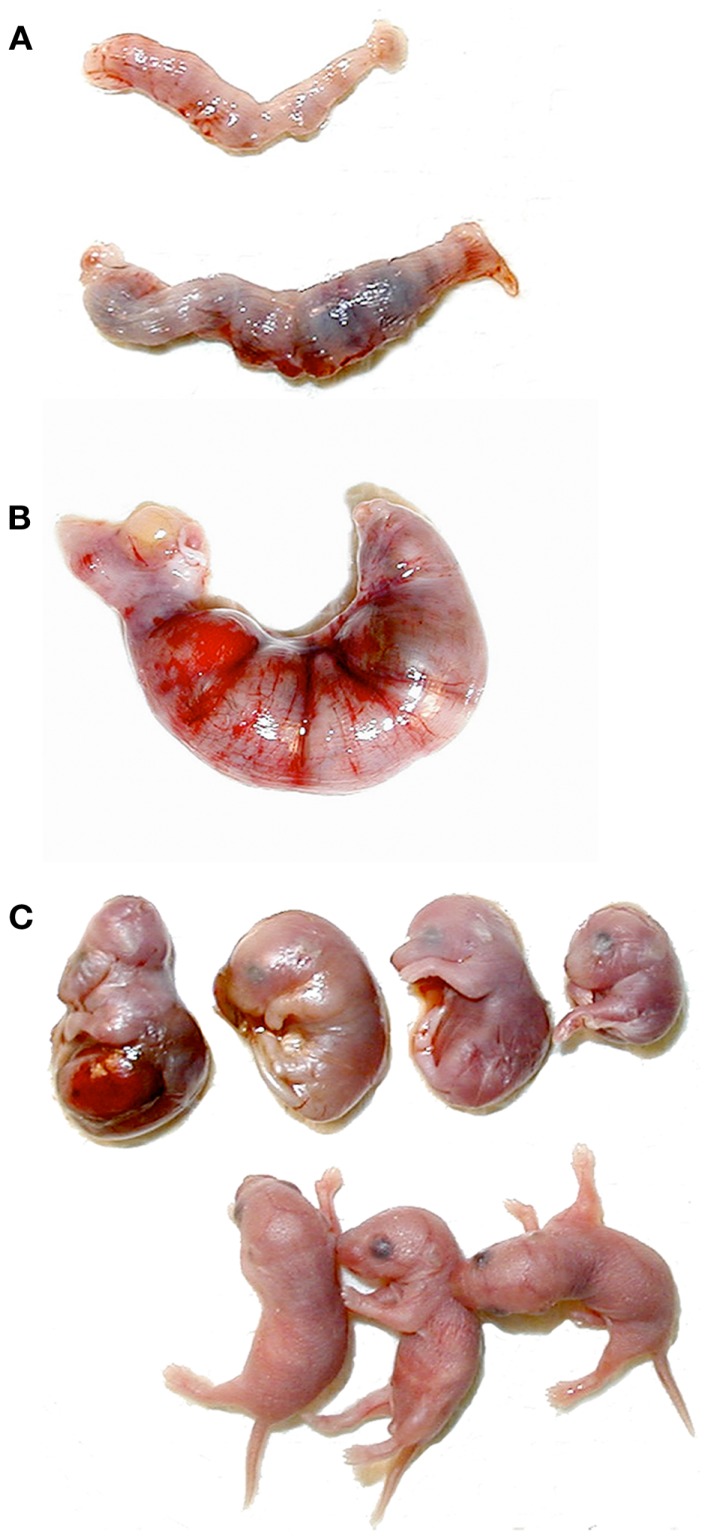
**Abnormal pregnancy outcomes in Clock mutant females**. **(A)** Uteri removed from postpartum wildtype (top) and *Clock/Clock* (bottom) females. The dark regions in the *Clock* mutant uterus are reabsorbing fetuses. **(B)** Uterus removed from a *Clock* mutant female after 24 h of dystocia. **(C)** Fetuses removed from the *Clock/Clock* uterus in **(B)**, shown with age-matched live fetuses from a wildtype dam (bottom).

Of the few *Clock* homozygotes who reached full term, half (as opposed to 0% of the wildtypes) displayed severe dystocia. Serum estradiol, progesterone, and glucocorticoid levels all regulate the timing and progression of parturition, but the specific cause of the parturition defects in *Clock* mutant mice has yet to be determined experimentally. Females who exhibited signs of unsuccessful labor for more than 24 h were sacrificed, and the uterine contents were examined. In all cases, the fetuses were clearly full term, but were dead and often exhibited gross morphological abnormalities (Figure 6). It is not known whether these abnormalities were the cause or the result of protracted labor, although it has been shown in rats that fetal death usually does not occur for at least 32 h after the onset of labor (68). This raises the possibility that fetal growth abnormalities may be more common in *Clock* mutants. In fact, the observed abnormalities are similar to those observed in wildtype mice nearing reproductive senescence (11–14 months of age), although the mice used in this study were <5 months old (69). Additionally, we have shown that mouse embryonic fibroblasts harvested from *Clock*/Clock mice exhibit a high rate of G1/S stage block, suggesting some difficulties with cell proliferation (70). Despite this evidence, when *Clock*/+ × *Clock*/+ matings are performed, there is no obvious imbalance in the expected percent of *Clock* homozygotes compared to either heterozygotes or wildtypes. Therefore, it is possible that defects in fetal growth and morphology are due to changes in the maternal hormonal milieu rather than a direct result of the Clock mutation on fetal development or maternal circadian rhythms.

## Summary and Relevance to Human Reproduction

Although it has long been known that multiple aspects of female reproductive exhibit circadian regulation, our understanding of the role that the central molecular circadian pacemaker plays has been substantially advanced by the availability of multiple circadian mutant mice. Dysregulation of core circadian genes, including *Clock, Bmal1*, and *Per1* and *Per2*, can disrupt the length and progression of the estrous cycle, the size and timing of the proestrus LH surge, the establishment and maintenance of pregnancy, and the success of parturition.

Human reproduction is similarly subject to circadian control. In women, the LH surge generally occurs immediately prior to the start of the active period, while the onset of parturition generally occurs during inactive period as a result of the circadian secretion of the pineal hormone melatonin (16, 71–74). Disruption of circadian rhythms due to rotating and night shift work or jet lag have been associated with an increase in the frequency of irregular, extended menstrual cycles, alterations in serum LH and FSH levels, an increased risk of pre-term birth, and overall reduced fecundity (75–77). Finally, polymorphisms in BMAL1 and the CLOCK paralog NPAS2 have been associated with the rate of pregnancy and risk of miscarriage (78). There is a long history of observational data among women, but a limited amount of molecular and genetic data to drawn upon.

Notably, a number of the defects exhibited by circadian mutant mice resemble phenotypes observed during the transition to reproductive senescence in middle-age wildtype mice. In a series of basic studies, Finch and colleagues found that wildtype mice exhibit the most regular estrous cycles from 3 to 10 months of age, after which cycle length increases and frequency decreases; the onset of persistent estrus, as evidenced by continuously cornified cells, occurs at 13–16 months (79). In our hands, young (2–5-month-old) *Clock/Clock* and *Bmal1* knockout mice show estrous cycle characteristics very similar to those described in middle-age wildtypes, and 7–8-month-old *Clock/Clock* females exhibit signs of persistent estrous similar to Finch’s 13–16-month-old wildtype mice. Pilorz and Steinlechner also noted that, while young Per1 and Per2 knockouts appeared normal, the knockouts exhibited an early onset of disrupted estrous cycles and pregnancy mice (19). Finally, although the *Clock* mutant dams described above were no more than 5 months old, the dystocia and fetal defects observed are similar to abnormalities described in 11–14-month-old wildtype mice (69). Thus, it is reasonable to consider alterations in central or peripheral pacemakers as a model for, or potential cause of, reproductive senescence.

Ultimately, molecular and genetic biology has confirmed long-standing observations regarding the role of circadian rhythms in female reproductive function. Almost all circadian mutant mice display reproductive defects to some extent, underlining the absolute importance that daily timing signals play in reproduction. These defects have multiple causes, not all of which are discussed here: in some cases, the daily timing signal needed to drive the LH surge is lacking, in other cases the clocks in peripheral reproductive organs are too disrupted to function properly, and in still other cases, the core circadian genes and transcription factors CLOCK and BMAL may alter the genetic landscape enough that reproduction fails.

## Conflict of Interest Statement

The authors declare that the research was conducted in the absence of any commercial or financial relationships that could be construed as a potential conflict of interest.
